# The Development of the Mesoprefrontal Dopaminergic System in Health and Disease

**DOI:** 10.3389/fncir.2021.746582

**Published:** 2021-10-12

**Authors:** K. Ushna S. Islam, Norisa Meli, Sandra Blaess

**Affiliations:** ^1^Neurodevelopmental Genetics, Institute of Reconstructive Neurobiology, Medical Faculty, University of Bonn, Bonn, Germany; ^2^Institute of Neuropathology, Section for Translational Epilepsy Research, Medical Faculty, University of Bonn, Bonn, Germany

**Keywords:** prefrontal cortex, innervation, dopamine receptors, neuropsychiatric diseases, ventral midbrain

## Abstract

Midbrain dopaminergic neurons located in the substantia nigra and the ventral tegmental area are the main source of dopamine in the brain. They send out projections to a variety of forebrain structures, including dorsal striatum, nucleus accumbens, and prefrontal cortex (PFC), establishing the nigrostriatal, mesolimbic, and mesoprefrontal pathways, respectively. The dopaminergic input to the PFC is essential for the performance of higher cognitive functions such as working memory, attention, planning, and decision making. The gradual maturation of these cognitive skills during postnatal development correlates with the maturation of PFC local circuits, which undergo a lengthy functional remodeling process during the neonatal and adolescence stage. During this period, the mesoprefrontal dopaminergic innervation also matures: the fibers are rather sparse at prenatal stages and slowly increase in density during postnatal development to finally reach a stable pattern in early adulthood. Despite the prominent role of dopamine in the regulation of PFC function, relatively little is known about how the dopaminergic innervation is established in the PFC, whether and how it influences the maturation of local circuits and how exactly it facilitates cognitive functions in the PFC. In this review, we provide an overview of the development of the mesoprefrontal dopaminergic system in rodents and primates and discuss the role of altered dopaminergic signaling in neuropsychiatric and neurodevelopmental disorders.

## Mesoprefrontal Dopaminergic Neurons

**FIGURE 1:**
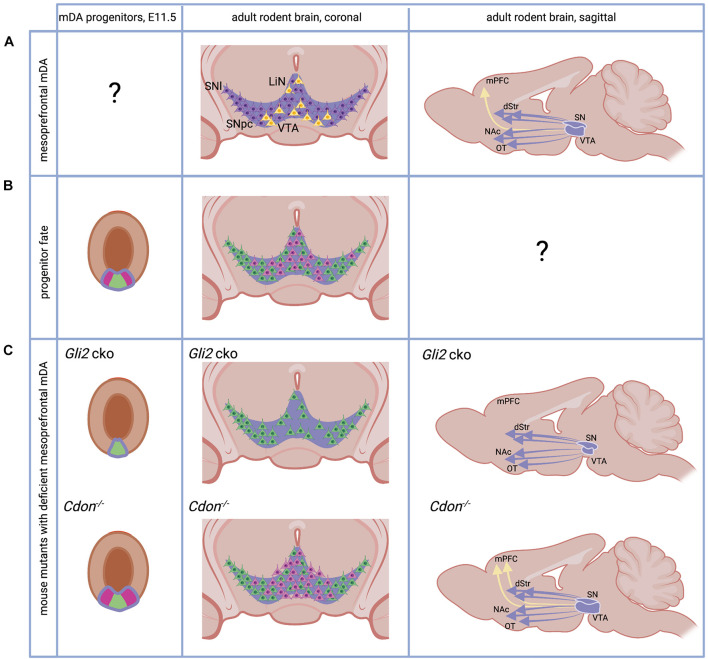
The adult and developing mesoprefrontal DA system in rodents. **(A)** Localization of mesoprefrontal mDA neurons (yellow) in the adult ventral midbrain (coronal view) and their projections (yellow arrow) to the adult medial PFC (mPFC, sagittal view). Non-mesoprefrontal mDA neurons and projections are in purple. Note that it is unknown whether there are specific mesoprefrontal mDA progenitors (indicated by “?”). **(B)** The mDA progenitor domain (purple outline) is divided in a medial (green) and lateral (pink) domain based on gene expression. Progenitors from these two domains give rise to mDA neurons with different anatomical location in the adult brain (pink and green neurons in coronal view). Note that it has not been examined whether mDA progenitors from these two domains form specific subcircuits in the DA system (indicated by “?”). **(C)** Mice with alterations in the SHH signaling pathway have an altered mesoprefrontal DA system. Conditional inactivation of GLI2 (*Gli2* cko) results in loss of the lateral progenitor domain, a reduced number of VTA neurons and loss of mesoprefrontal DA projections. Inactivation of CDON (*Cdon*^–/–^) results in increased proliferation of mDA progenitors, an increased number of VTA neurons and increased DA release in the mPFC. See main text for details. dStr, dorsal striatum, NAc, nucleus accumbens, OT, olfactory tubercle. Created with BioRender.com.

Midbrain dopaminergic (mDA) neurons modulate many brain functions including voluntary movement, reward behavior, and cognitive processes (Iversen et al., 2009). Degeneration of a subset of mDA neurons underlies the motor deficits in Parkinson’s disease, while altered dopamine (DA) transmission is implicated in neuropsychiatric disorders including depression, schizophrenia, autism, ADHD, and substance abuse (Del Campo et al., 2011; Volkow and Morales, 2015; Grace, 2016; Surmeier et al., 2017; Marotta et al., 2020; Sonnenschein et al., 2020). mDA neurons are located in the ventral midbrain where they form the A8, A9, and A10 group. The A10 neurons are located in the ventral tegmental area (VTA) and linear nucleus (LiN), the A9 neurons in the substantia nigra pars compacta (SNpc) and substantia nigra pars lateralis (SNl), while the A8 group is found in the retrorubral field (RRF). mDA neuronal projections run through the medial forebrain bundle (MFB) and then diverge into the various forebrain target areas, including dorsal striatum, amygdala, nucleus accumbens, olfactory tubercle, and prefrontal cortex (PFC) (Iversen et al., 2009; Figure 1A). In recent years, molecularly distinct mDA subpopulations as well as anatomically and physiologically discrete DA circuits and their effects on various aspects of behavior have been studied in increasing detail, driven by rapid advances in single-cell gene expression profiling, viral tracing systems, DA sensors, and opto- and chemogenetic techniques (e.g., Lammel et al., 2011; Beier et al., 2015; Menegas et al., 2018; Poulin et al., 2018, 2020; Saunders et al., 2018; Engelhard et al., 2019; Lee et al., 2021). Based on these and numerous other studies, it is now evident that the DA system is composed of diverse populations of mDA neurons and that this diversity is critical for the various functional performances of the DA system. In this review, we focus specifically on the mesoprefrontal DA system, which is formed by mDA neurons that project to the PFC.

In the adult rodent brain, mesoprefrontal mDA neurons are primarily localized in the medial and ventral VTA region and LiN (Lammel et al., 2011; Yamaguchi et al., 2011; Figure 1A). These mesoprefrontal mDA neurons differ in their molecular profile (e.g., express low levels of dopamine transporter) and in their electrophysiological properties from other mDA neurons, indicating that they form a distinct subclass of mDA neurons (Lammel et al., 2011). This is supported by tracing studies in rodents that show that mesoprefrontal mDA neurons do not send extensive collaterals to other forebrain areas (Aransay et al., 2015; Beier et al., 2019). On a functional level, it has been demonstrated that aversion is encoded by mesoprefrontal mDA neurons while mDA neurons projecting to the nucleus accumbens encode reward. These distinct functions are associated with distinct inputs: aversion-encoding mesoprefrontal mDA neurons receive inputs from the lateral habenula, while the reward-encoding mDA neurons are activated by inputs from the lateral-dorsal tegmentum (Lammel et al., 2012). It is important to note that a substantial fraction of these mesoprefrontal mDA neurons co-express *Slc17a6* (the gene encoding the vesicular glutamate transporter 2, vGLUT2) indicating that they have the ability to co-release the neurotransmitter glutamate (Yamaguchi et al., 2011; Poulin et al., 2018). In the primate brain, the results of a recent viral tracing study in macaques suggest that mDA neurons in the medial VTA may be the main source of DA innervation to the PFC, whereas lateral VTA or medial SNpc mDA neurons are more likely to send projections to motor and somatosensory cortices (Zubair et al., 2021). An analysis of *SLC17A6* expression in marmosets and humans demonstrates that mDA neurons in the lateral VTA and LiN co-express vGLUT2 also in primates, but whether these co-expressing cells are part of the mesoprefrontal DA system is unknown (Root et al., 2016).

At the functional level, decades of research have shown that the mesoprefrontal DA system exerts a profound modulatory function on the PFC and strongly influences PFC-mediated executive functions (i.e., working memory, decision making, behavioral flexibility) and PFC-regulated behaviors (goal-directed behavior, approach-avoidance behavior, response to stress or pain). Since the focus of this review is the development of the mesoprefrontal system, we refer the interested reader to some recent reviews covering the functional aspects of the mesoprefrontal DA system (Weele et al., 2018; Pastor and Medina, 2021; Starkweather and Uchida, 2021).

## Prefrontal Cortex in Rodents and Primates

Before discussing the organization of the mesoprefrontal system and its development in more detail, we will briefly describe how we define the terms PFC and medial PFC (mPFC) in rodents and primates in the context of this review. There is still no consensus on what constitutes the PFC, especially since there is disagreement regarding the subdivisions of prefrontal cortical areas in different species. Functionally, the human PFC is subdivided into dorsolateral, dorsomedial, ventrolateral, ventromedial, and orbital prefrontal cortex. These areas are mostly granular, showing a six-layered laminar organization with a distinct granular layer IV. However, some parts of the primate PFC consist of dysgranular cortex with an indistinct layer IV or agranular cortex in which layer IV is completely absent, such as the anterior cingulate cortex. In contrast, all frontal cortical areas are agranular in rodents, thus lacking the subdivision into granular and dysgranular cortices (Carlén, 2017; Laubach et al., 2018). Nevertheless, functional data suggest that the prelimbic, infralimbic, and anterior cingulate cortices of rodent frontal cortex have functions that are attributed to the dorsolateral PFC and anterior cingulate cortices in primates (Uylings et al., 2003; Seamans et al., 2008). These regions are classified as prefrontal in rodents. Because these areas are located in the medial frontal cortex in both rodents and primates, they are referred to as the mPFC (Laubach et al., 2018). We therefore use the term mPFC to describe the prelimbic, infralimbic, and anterior cingulate cortex in rodents. The cingulate cortex that extends from the genu of corpus callosum caudally, the anatomical region immediately posterior to the mPFC, is referred to as caudal cingulate cortex in our review. For studies in primates and rodents in which the prefrontal subregions are not specified in terms of the above definitions, we followed the terminologies used in the original publications.

## Development of the Prefrontal Cortex

The cerebral cortex exhibits an orderly laminar organization that is established during embryonic development. While the PFC is the last cortical area to fully mature in terms of inputs and local microcircuits, there is no clear evidence that the timing of early cortical development (neurogenesis, layer formation) is markedly different from other cortical areas. Two recent reviews have discussed in detail the development of the PFC in anatomical and functional terms (Schubert et al., 2015; Chini and Hanganu-Opatz, 2020). The basic steps of corticogenesis are summarized in Supplementary Figure 1.

In the next paragraphs, we will focus on the development of the mesoprefrontal DA system in rodents and primates. For a detailed account of the general development of the rodent DA system see the following reviews (Blaess and Ang, 2015; Brignani and Pasterkamp, 2017; Ásgrímsdóttir and Arenas, 2020).

## The Dopaminergic Progenitor Domain – Specific Progenitors for Mesoprefrontal dopaminergic Neurons?

Midbrain dopaminergic neurons develop from progenitors in the floor plate of the ventral midbrain. The floor plate, located in the ventral midline of the neural tube, is different from the surrounding neuroepithelia tissue in the neural tube since: (1) its lineage diverges from the neuroepithelia fate quite early, and (2) it serves as one of the organizing centers in the development of the midbrain, by secreting the ventralizing factor Sonic Hedgehog (SHH) (Bodea and Blaess, 2015). The expression of *Shh* in the midbrain floor plate is dynamic (Joksimovic et al., 2009; Blaess et al., 2011; Hayes et al., 2011). Initially, around E8.0 in mice, *Shh* is expressed only in the notochord, a mesodermal structure underlying the ventral neural tube. Cells in the midline of the forming neural tube respond to SHH signaling. This response can be visualized by the presence of *Gli1*, a transcription factor in the SHH signaling pathway only expressed in cells that receive high levels of SHH signaling. SHH-responding cells are specified into floor plate cells, characterized by the expression of the transcription factor FOXA2 (Forkhead box A2). The FOXA2-positive floor plate cells stop responding to SHH signaling but start to secret SHH themselves and induce floor plate fate in neighboring cells. This process continues until E10.5, when the middle third of the ventral midbrain has been transformed into FOXA2-expressing cells. Within the floor plate domain, the medial area expresses the transcription factor LMX1A (LIM homeobox transcription factor 1 alpha) and this is the region that eventually gives rise to mDA neurons (Andersson et al., 2006; Figure 1B). This LMX1A-expressing domain can be further subdivided into a medial and lateral domain based on gene expression. For example, it has been shown that OTX2 (Orthodenticle Homeobox 2) and NOLZ1 (also known as ZNF503) are restricted to the lateral domain, while SOX6 (sex determining region Y (SRY)-box 6) is expressed in medial progenitors (Panman et al., 2014; Figure 1B). Fate-mapping studies of medial and lateral domain progenitors come to conflicting results about their contribution to different anatomical domains of the DA system in the adult brain (Poulin et al., 2020), but several lines of evidence suggest that the medial progenitor domain is biased to give rise to neurons of the SNpc and the lateral VTA while the lateral progenitor domain gives rise to the medial VTA (Blaess et al., 2011; Hayes et al., 2011; Panman et al., 2014; Figure 1B). SHH signaling is essential for the induction of the mDA progenitor domain, but SHH signaling is required longer for induction of the lateral progenitor domain than for induction of the medial domain. This is evident from *Gli1* expression, the above-mentioned readout for high-level SHH signaling, which is downregulated first in the medial and then in the lateral domain. Thus, conditional inactivation of the transcription factor GLI2 downstream of the SHH pathway in the midbrain around E8.5 (*Gli2* conditional ko mice) essentially abolishes SHH signaling activity in the ventral midbrain. Since the medial domain no longer requires SHH for its induction at this time point, it is formed, albeit at a smaller size. In contrast, the lateral mDA progenitor domain is almost completely absent. In the brain of adult *Gli2* conditional ko mice, the number of mDA neurons in the medial VTA is severely reduced and projections to the mPFC are absent, while projections to other VTA or SNpc target areas are not overtly reduced (Kabanova et al., 2015; Figure 1C). Interestingly, inactivation of the gene encoding CDON (Cell adhesion molecule-related/downregulated by oncogenes), a co-receptor of the SHH receptor Patched 1 that modulates SHH pathway activity and is expressed in mDA progenitors, leads to the opposite result: the number of proliferating mDA progenitors is increased and so is the number of mDA neurons in the VTA in the adult brain. The number of mDA neurons in the SN is not significantly altered. The increase in VTA-mDA neurons goes along with increased DA release and a higher number of DA presynaptic sites in the mPFC, an effect that is not observed in other target areas of the VTA (Verwey et al., 2016; Figure 1C). Importantly, the function of SHH signaling in cell fate specification in the ventral midbrain can be largely pinpointed to its role in mDA progenitors. GLI transcription factors, which are essential for SHH downstream signaling, are not expressed in differentiated mDA neurons and accordingly *Gli1*, the readout for the activated pathway, is not detected in differentiated mDA neurons (Mesman et al., 2014). In summary, these studies suggest that SHH signaling is required after E8.5 in the developing mouse brain to induce the lateral mDA progenitor domain and that this domain contains the progenitors that give rise to mesoprefrontal mDA neurons.

## Differentiation Onset of Midbrain Dopaminergic Neurons - Late Birth Date of Mesoprefrontal dopaminergic Neurons?

**FIGURE 2:**
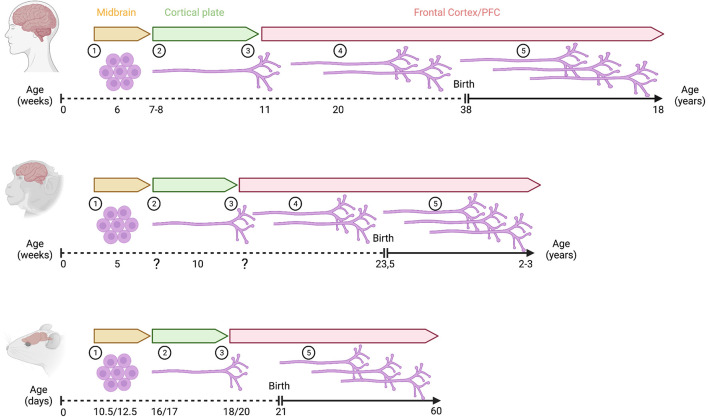
Development of mesoprefrontal DA projections in primates and rodents. Critical developmental stages of the mesoprefrontal DA system are shown as follows: (1) onset of differentiation of dopaminergic neurons, (2) dopaminergic axons reach the cortical region, but do not yet enter into the developing cortical plate (3) dopaminergic axons innervate the cortical plate, (4) density of innervation increases during embryonic development (indicated by multiple axons), (5) density of innervation increases further during postnatal development (indicated by multiple axons). Note that the increase in innervation density occurs essentially only during the postnatal period in rodents. In primates, the timeline of prenatal development is shown in weeks, and the postnatal period is shown in years. In rodents, prenatal and postnatal stages are indicated in days (mouse/rat). Created with BioRender.com.

In mouse, cell cycle exit of mDA neurons starts at around E10 and continues until about E14.5 (Bayer et al., 1995; Bye et al., 2012). Expression of tyrosine hydroxylase (TH), the rate limiting enzyme of the DA synthesis pathway is first observed between E10 and E10.5 (Dumas and Wallén-Mackenzie, 2019). Besides the evidence for spatial distinct progenitor domains described in the previous paragraph, there is also evidence that specific mDA subpopulations differ in their birth date (i.e., differentiation onset). In mice, the peak of cell cycle exit occurs earlier for mDA neurons of the SNpc (around E10.5) than for the ones forming the VTA (around E11.5). This peak is shifted to an even later time point (E13.5) for the interfascicular nucleus in the ventromedial VTA (Bayer et al., 1995; Bye et al., 2012). A similar temporal sequence in mDA differentiation onset has been described in rat: SNpc neurons are born between E12.5 and E15.5, with a peak at E12.5; mDA neurons of the lateral VTA are born in the same period but with a peak at E13.5; and those of the medial VTA are generated between E13.5 and E16.5 with a peak around E15.5 (Altman and Bayer, 1981). Since mesoprefrontal mDA neurons are mostly located in the medial and ventral VTA in rodents (Yamaguchi et al., 2011), this could suggest that these neurons are born later than other mDA neurons. In primates, the development of the catecholaminergic system starts early in embryonic development and the onset of SNpc neuron generation is also earlier than the one for VTA neurons. In rhesus monkey, mDA neurons are detected during the first quarter of gestation [5–6 gestational weeks (gw)]. mDA neurons in the SNpc are generated first, between E36-E43, followed by mDA neurons in the VTA (E38-E43) (Levitt and Rakic, 1982). In humans, distinct TH-expressing cell populations can be detected along the rostrocaudal axis of the brain already at 6 gw (Freeman et al., 1991; Verney et al., 1991; Zecevic and Verney, 1995). At this stage, prominent regions with dense clusters of TH-expressing cells are found in the mesencephalon probably representing the anlage of the three different midbrain mDA groups: A8 caudally, A9 laterally, and A10 medially (Figure 2). Generally, the sequence of these early events in the developing DA system in rodents and primates are remarkably similar. However, the timing of these events is not synchronized across these species, considering their respective gestational lengths. Based on a study that equates neurodevelopmental stages across mammalian species (Clancy et al., 2001), 6 gw in humans and 5 gw in macaques are considered earlier gestational timepoints than E10.5 in mice and E12.5 in rats (Supplementary Figure 1). Thus, the first appearance of TH-expressing neurons seems to occur earlier in primates than in rodents.

While these rodent and primate data indicate that mDA neurons in SNpc, lateral, and medial VTA differ in their onset of differentiation, there is as yet no clear evidence that birth date also correlates with mDA subpopulations with specific projection targets (e.g., in mice, are all mesoprefrontal mDA neurons born after E13.5, or all mDA neurons projecting to the nucleus accumbens born before E13.5?). Moreover, it is not known whether mesoprefrontal mDA neurons (and other mDA subpopulations defined by their projection targets) can be characterized by a particular gene expression profile (Poulin et al., 2020). *Slc17a6*, the gene encoding vGLUT2, is expressed in a subset of mesoprefrontal mDA neurons in the adult rodent brain but is not in itself a marker for this subset, as it is also expressed in a subpopulation of nucleus accumbens-projecting VTA-mDA neurons and in SNl-mDA neurons projecting to the tail of the striatum (Yamaguchi et al., 2011; Poulin et al., 2018). Interestingly, *Slc17a6* is broadly expressed in mDA neurons during development and only gets restricted to the above-mentioned mDA subtypes in the postnatal brain (Steinkellner et al., 2018; Dumas and Wallén-Mackenzie, 2019; Kouwenhoven et al., 2020). The expression of *Slc17a6* in mesoprefrontal mDA neurons in the adult mouse brain is consistent with data showing that a subset of these mDA neurons co-release glutamate in the PFC. This glutamate release primarily leads to the excitation of cortical interneurons (Kabanova et al., 2015; Mingote et al., 2015; Pérez-López et al., 2018; Zhong et al., 2020).

This restricted effect of mDA-mediated glutamate release on GABAergic interneurons, and in particular on a subset of fast-spiking interneurons, could contribute to the refinement of local PFC circuit function. One important component of the protracted functional remodeling process of the PFC during postnatal development is the maturation of these local circuits. This is thought to be largely driven by the maturation of GABAergic interneurons. These changes ultimately lead to the fine-tuning of the excitatory–inhibitory balance in the PFC, which is essential for its normal function (Caballero and Tseng, 2016). Rapid activation of GABAergic interneurons by mDA-mediated glutamate release could lead to the rapid inhibition of projection neurons in the PFC and regulate the sparseness and precision of their activation, thus acutely modulating the excitatory–inhibitory balance in PFC neuronal networks. In contrast, the long-term processing dynamics of local circuits in the PFC could be modified by the long-lasting effect of DA. This target specificity of the glutamate effect is consistent with the results of a study in which it was shown that electrical stimulation in the VTA leads to glutamate-dependent feed-forward activation of interneurons in the PFC, whereas a form of DA-induced potentiation occurs over a much longer period (Lavin et al., 2005).

## Development of the Mesoprefrontal Dopaminergic Projections in Rodents

Several studies in rodents have followed the development of mDA projections and innervation of their forebrain targets by means of antibody labeling, directed either against DA or TH. Before we start to describe the development of mDA fibers in the PFC, it is necessary to briefly discuss the expression of DA and TH, the primary markers that have been used for this analysis. TH is the rate-limiting enzyme in DA synthesis and thus a marker for mDA neurons. Since DA is the direct precursor of noradrenaline, TH and DA are also present in noradrenergic (NA) neurons. Thus, TH and DA are markers for both DA and NA neurons. Since mDA neurons and NA neurons from the locus coeruleus send projections to the PFC (Levitt and Moore, 1979), TH or DA staining in the PFC should in principle detect both DA and NA axons. However, double immunohistochemistry for TH and Dopamine beta-hydroxylase (DBH, a specific marker for NA axons) in the prefrontal areas of adult human brain shows that approximately 15% of DBH-positive axons are also co-labeled with TH. In fetal brains, the overlap is even lower (ca. 5%) (Gaspar et al., 1989; Verney et al., 1993). These data suggest that, at least in humans, both during development and in the adult brain, TH immunoreactivity in axonal fibers is largely restricted to projections from mDA neurons. Nevertheless, when drawing conclusions from studies using TH or DA as markers for mDA projections in the PFC, it should be kept in mind that NA fibers may also be labeled to a certain extent.

In the adult rodent mPFC, there is a dense input of TH-positive fibers to the deep layers, while innervation of TH-expressing fibers is much sparser in the superficial layers of the mPFC, except for the caudal cingulate cortex, which also has dense TH-positive innervation in layers I-III (Kalsbeek et al., 1988; Naneix et al., 2012).

In the developing rodent brain, TH immunoreactivity reveals that mDA neurons in the ventral midbrain of rodents start to extend axonal processes between E11 and E12. In mice, axons initially grow slightly dorsally, but by E13, almost all axons follow a rostral course and by E13.5 form a TH-positive axon tract within the MFB, which is directed toward forebrain targets (Nakamura et al., 2000; Kolk et al., 2009). One day later in development, the TH-positive fiber tract reaches a region ventral to the ganglionic eminences (Kolk et al., 2009). Analysis of DA-positive fiber bundles in rats showed that they reach this region also around E14 (Kalsbeek et al., 1988; Voorn et al., 1988). In mice, while most of the TH-positive axons from the MFB begin to move dorsally to innervate the maturing striatum, a small number of fibers follows a rostrodorsal trajectory towards the frontal cortex. These TH-positive axons follow two paths to reach the mPFC. The larger TH bundle bends just before the olfactory bulb and extends toward the cortical subplate, while the smaller subset of TH axons passes through the striatum to the developing mPFC. The TH-positive fibers arrive in the subplate and marginal zone around E15 and continue to grow for about 2 days without entering the cortical plate, which develops and enlarges in the meantime. At E18.5, the first TH-positive axons are detected in the cortical plate (Figure 2). Tracing experiments with the lipophilic fluorescent dye DiI show that after microinjection of DiI into the mPFC at E16.5 and postnatal day (P)0, the dye is eventually detected in the rostral VTA. Conversely, after DiI microinjection into the rostromedial VTA, DiI-stained, TH-positive axons are found in the subplate at E16 and in the cortical plate at E18.5. However, no DiI-stained fibers are found in the marginal zone of the PFC in the latter experiment. Together, these data suggest that one subset of mesoprefrontal projections in mice originates in the rostral medial VTA, while a second subset originates from mDA neurons in another ventral midbrain region (Kolk et al., 2009). In rats, the TH-positive axons within the MFB also arrive in the mPFC in two separate bundles. At E18, one of the axonal bundles is observed above the subplate while the other axonal trail can be detected within the marginal zone. The DA fibers in the future mPFC adopt a coiled structure and start innervating the thickening cortical plate from E20 onwards (Kalsbeek et al., 1988; Kolk et al., 2009; Garcia et al., 2019).

Shortly after birth, DA-positive fibers in rats are primarily located in the developing layer VI of mPFC, orbital cortex, and caudal cingulate cortex (defined as supragenual mPFC in the original study by Kalsbeek and colleagues). At P2, the fiber density in layer VI increases substantially. Between P2 and P4, the DA axons change their morphology from thick, straight fibers to thin fibers with irregularly shaped varicosities. This marks the beginning of postnatal maturation of DA-positive fibers in the mPFC, which continues into early adulthood. By the end of the first postnatal week, the infralimbic subdomain of the mPFC shows already an adult-like pattern of DA innervation, with DA-positive fibers reaching up to the pial surface. In other areas of the mPFC, only a few DA-positive fibers in layer I are detectable at this developmental stage. The density of DA fibers in the deeper layers continues to increase in the second postnatal week. At P20, DA-positive projections reach the upper cortical layers II and I in the prelimbic cortex. At this stage, the DA-positive fibers in layer I of the anterior cingulate cortex of mPFC fade away, but the projections in the caudal cingulate cortex are found in layers II and III. The morphological characteristics of DA-positive fibers in the mPFC, with thin axons and multiple varicosities, do not change significantly after P35, but the density of fibers continues to increase until adulthood, with the deeper layers becoming more densely innervated than the upper layers (Kalsbeek et al., 1988). TH immunostaining in rat mPFC shows that the increase in TH-positive fibers is relatively rapid during adolescence, whereas the density of DBH-expressing NA fibers in mPFC remains constant from early adolescence to adulthood (Naneix et al., 2012; Willing et al., 2017) (Figure 2). The delayed developmental trajectory of prefrontal TH-positive axons from early adolescence to adulthood is similar in male and female rats, even though pubertal onset is approximately 10 days earlier in female than in male rats. These data indicate that sex or pubertal onset do not affect the maturation profile of mesoprefrontal innervation (Willing et al., 2017).

In addition to the innervation density, the formation of varicosities on DA fibers and thus potential release sites is likely another important indicator of functional maturation of DA fibers. DA immunoreactive varicosities have been found to form appositions with both pyramidal and nonpyramidal somata in the mPFC. This is especially noticeable in layer VI, where the density of DA varicosities is higher and GABA-positive cell bodies are frequently found to be in close contact with DA varicosities (Benes et al., 1993). The number of close appositions formed by GABA-positive cell bodies with DA varicosities shows a steady increase from P5 to P60, while the number of varicosities closely interacting with each GABA-positive neuron increases more rapidly during the postweaning period (P25–P59) to reach young adult levels (P60) (Benes et al., 1996).

In mice, the change in TH/DA fiber density in the mPFC during the juvenile and adolescent periods has not yet been studied in detail. To gain insight into potential mechanisms underlying protracted DA innervation of the mPFC, Reynolds and colleagues used an elegant virus-based approach to axon labeling. In this study, retrogradely transported canine adenovirus (CAV) expressing Cre recombinase was injected into the nucleus accumbens of mice during early adolescence (P21), whereas a virus expressing a fluorescent protein after Cre-mediated recombination was injected into the VTA. CAV-Cre is taken up by axon terminals in the nucleus accumbens, so that only VTA neurons whose axons have reached the nucleus accumbens around P21 are fluorescently labeled. The authors then showed that fluorescently labeled fibers are present in the mPFC of adult mice. These results indicate that the late maturation of DA fibers in the mPFC may be due to at least some of the fibers initially innervating the nucleus accumbens and only projecting into the mPFC during later stages of adolescence (Reynolds et al., 2018).

Directing the extending DA axons to their proper targets requires precise coordination of extracellular axon guidance cues, receptor complexes, cell adhesion molecules, neurotrophic and growth factors (Hoops and Flores, 2017; Vosberg et al., 2020). Several guidance cue pathways involved in regulating the axonal pathfinding of mesoprefrontal DA axons have been identified. This includes Ephrins, Slits, Semaphorins, Netrins and their receptors. During early stages of mDA development, Semaphorin 3F acts via its receptor Neuropilin-2 to repel mDA axons away from the midbrain, while it changes its role into a chemoattractant to guide the DA axons towards the cortical plate of the mPFC at the prenatal stage (Kolk et al., 2009). The extracellular protein Netrin-1 and its receptor, DCC (deleted in colorectal cancer) also play a key role in mesoprefrontal/mesolimbic axon growth and the fine-tuning of their expression levels during adolescence is critical to help DA axons find their final target (Reynolds et al., 2018). We will not discuss these molecular mechanisms further here, as they have been extensively addressed in two recent reviews (Brignani and Pasterkamp, 2017; Hoops and Flores, 2017).

## Development of the Mesoprefrontal Dopaminergic Projections in Primates

In the adult primate brain, the densest TH-positive innervation is observed in primary motor cortex rather than in PFC areas (Gaspar et al., 1989; Raghanti et al., 2008). While primary motor cortex (area 4) shows even distribution of TH-positive fibers across all layers in the human brain, the PFC shows a bilaminar distribution with highest innervation density in layer I and V–VI (area 9 and 32) (Gaspar et al., 1989; Raghanti et al., 2008). Such bilaminar innervation was not detected in adult non-human primate PFC (Lewis and Harris, 1991; Rosenberg and Lewis, 1995; Raghanti et al., 2008). On an ultrastructural level, electron microscopy of DA axonal boutons (marked with antibodies against DA and TH) in the PFC of rhesus monkey shows that they form symmetric synaptic connections with dendritic spines of pyramidal cells (Goldman-Rakic et al., 1989). In addition, DA afferents also contact dendrites of nonpyramidal inhibitory interneurons in rhesus monkey PFC (Smiley and Goldman-Rakic, 1993).

How does this innervation pattern develop? Similar to rodents, primate mesoprefrontal DA fibers undergo a protracted development that may involve reorganization of innervation density until the functionally mature innervation pattern of the adult brain is established (Gaspar et al., 1989; Raghanti et al., 2008). In rhesus monkey, TH-expressing axons are observed in the cortical anlage during the 10th gw (Verney, 1999). In neonatal rhesus monkeys, the TH positive innervation is bilaminar in the PFC (area 9), similar to the pattern in the adult human brain. TH positive axons in the rhesus monkey PFC are reorganized from birth till adulthood, resulting in the relatively uniform distribution of TH positive innervation across layers in the adult PFC (Lewis and Harris, 1991; Rosenberg and Lewis, 1995). Accordingly, it is the innervation of intermediated cortical layers (especially layer III) that increases with age and reaches its peak in 2-3-year-old adolescent rhesus monkeys (Rosenberg and Lewis, 1995). Based on the observation that the direct effect of DA on the spontaneous activity of PFC neurons is mostly inhibitory, the increased TH positive innervation in layer III of adolescent PFC might indicate an increase in a DA-mediated inhibitory effect onto the pyramidal neurons in these layers (Rosenberg and Lewis, 1995; Figure 2).

In humans, TH-expressing neurons are detected as early as 6 gw and already extend processes that eventually give rise to the mesencephalic tract. This tract, along with the dorsal tegmental bundle, forms the MFB (Zecevic and Verney, 1995; Verney, 1999). TH-positive fibers enter the telencephalic wall at 7-8 gw but remain below the cortical plate (intermediate and subplate area) for 4 weeks before they enter the cortex (Zecevic and Verney, 1995). At 20-24 gw, DA innervation is observed in the frontal cortex with a higher density of TH positive innervation in the anterior cingulate and motor area compared to the rostral prefrontal cortical anlage (Verney et al., 1993). It is interesting that this area-specific distribution and density of TH-expressing fibers at this stage is similar to what has been reported in the adult cortex (Gaspar et al., 1989; Verney et al., 1993; Verney, 1999), suggesting that the DA innervation pattern is in principle established already during fetal development in the human brain and subsequently only increases in density. Eventually, the adult PFC acquires its distinctive bilaminar innervation pattern (Gaspar et al., 1989; Raghanti et al., 2008; Figure 2).

Similar to the timing of differentiation onset of mDA neurons in rodents and primates, the outgrowth of TH-positive fibers and frontal cortex innervation also seems to occur earlier in humans than in rodents as 11 gw in humans is considered a much earlier gestational timepoint than E18 or E20 in mice and rats, respectively (Clancy et al., 2001; Figure 2 and Supplementary Figure 1).

## Dopamine Release and Dopamine Receptors in the Developing Prefrontal Cortex

While the location and density of DA projections gives some indication about when and where mesoprefrontal mDA neurons may modulate PFC function, the functional relevance of these projections can only be fully assessed by insights into actual DA release, DA receptor (DRD) expression, and the response of receiving cells to the DA release. In addition, as discussed previously, the release of neurotransmitters other than DA (most prominently glutamate) is likely to contribute to the functional output of the mesoprefrontal mDA neurons.

### Dopamine Release

Analysis of DA and its metabolites in rat mPFC by high throughput liquid chromatography (HPLC) showed that DA concentrations were significantly lower in juvenile and adolescent rats than in adults. DA concentration rose steadily between the juvenile (P25) and late adolescent stages (P45) and increased particularly sharply between the end of adolescence and adulthood. In parallel, a decrease in DA turnover ratios was observed with increasing age, an effect that could contribute to the overall increase in DA availability in the mPFC (Naneix et al., 2012). Analysis of DA tissue concentrations in rhesus monkey PFC showed that DA levels fluctuated between 2, 5, 8 and 15-18 months old animals and significantly increased in 2–3 years old animals (Goldman-Rakic and Brown, 1982). These data suggest that both in rats and rhesus monkey, the overall DA concentration coincides with the increase in DA fiber innervation of the PFC. However, whether this increase in concentration correlates with active DA release has not been investigated in the developing PFC. The recent development of genetically encoded DA sensors that allow the monitoring of DA release in the behaving animal, offer the opportunity to correlate behavior, PFC function and DA release in real-time in adolescent and adult animals (Labouesse et al., 2020).

### Dopamine Receptors and Downstream Signaling

Once released from the axonal varicosities of DA axons, DA binds to DA receptors (DRDs) of the D1-like or D2-like subfamily of G-protein coupled receptors. DRD1 and DRD5 belong to the D1-like subfamily, while DRD2, DRD3, and DRD4 are subtypes of the D2-like subfamily. Unlike *Drd1* and *Drd5*, the D2-like subfamily receptor genes contain introns that allow differential splicing of the transcripts, generating additional isoforms. *Drd2* comes in two alternatively spliced variants, Drd2s (short form) and Drd2l (long form), and isoforms of *Drd3* and *Drd4* have also been identified (Missale et al., 1998). D1-like receptors signal by coupling to G proteins G_a__s_ and G_a__olf_, which stimulate adenylyl cyclase and lead to activation of protein kinase A (PKA). D2-like receptors stimulate G_a__i_ and G_a__o_ proteins, blocking adenylyl cyclase and consequently inhibiting PKA activity (Missale et al., 1998; Tritsch and Sabatini, 2012). Furthermore, DRDs can activate a signaling cascade by interacting with ß-arrestin (Beaulieu et al., 2005) or induce phospholipase C-mediated increase of intracellular calcium levels (Lee et al., 2004), although the signal transduction pathway of this modulation remains to be resolved (Chun et al., 2013). The striatum and the nucleus accumbens receive dense projections from mDA neurons and have high expression levels of DRDs. In the PFC, the expression levels of the DRDs are considerably lower, correlating with relatively sparse innervation by DA fibers.

### Dopamine Receptor Expression in Rodent Prefrontal Cortex

The distribution and expression of DRDs and their transcripts in rodent PFC have been studied using multiple histological methods, real-time quantitative PCR and in recent years, genetic tools and single-cell transcriptome analysis (Table 1). Early studies include autoradiographic experiments employing radiolabeled agonist or antagonist of DRDs (Boyson et al., 1986; Noisin and Thomas, 1988), immunohistochemical and immunoblotting approach targeting the receptor protein (Levey et al., 1993; Sesack et al., 1994) and in-situ hybridization technique detecting *Drd* transcripts (Gaspar et al., 1995). Some of the radioligands used in binding assays were later found to lack selectivity for specific subtypes of DRD (Landwehrmeyer et al., 1993) and similar doubts have been expressed for commercially available antibodies for the receptors (Bodei et al., 2009). RNA in situ hybridization methods have characterized the distribution of certain *Drd* mRNAs within the subregions of the PFC (Santana and Artigas, 2017) and RT-qPCR approaches were used to quantify the relative gene expression of the *Drd* subtypes in the PFC (Araki et al., 2007). Whether the transcript levels reliably correspond to the expression levels of DRD protein is not known.

**TABLE 1:** Laminar distribution of *Drds*/DRDs in the PFC of rodent, rhesus monkey and human.

	Receptor / Gene	Rodent						Human / Non-human Primate								
		L2/3	L5	L6	Species	Method	References	L1	L2	L3	L4	L5	L6	Species	Method	References
D1-like Family	DRD1 */ Drd1*	++	++	+++	Rats	*In situ*	Santana and Artigas, 2017	(*+*)	+++	*++*	*++*	+++	+++	Humans	*In situ*	Weickert et al., 2007
		++	+++	+++	Rats	Receptor binding	Vincent et al., 1993									
		++	++	+++	Mice	Genetic labeling	Wei et al., 2018	+++	*+++*	*+++*	*++*	*+++*	*+++*	Rhesus Monkeys	Receptor Autoradiography	Lidow and Rakic, 1992
	DRD5 */ Drd5*	++	++	++	Mice	Immuno histochemistry	Lidow et al., 2003									
		+++	++	++	Rats	Immuno histochemistry	Ciliax et al., 2000									
D2-like Family	DRD2 */ Drd2*	+	+++	++	Rats	*In situ*	Santana and Artigas, 2017	(+)	*++*	*+*	*+*	*+++*	*+++*	Humans	*In situ*	Weickert et al., 2007
		++	+++	+++	Rats	Receptor binding	Vincent et al., 1993									
		+	+++	++	Rats	Genetic labeling	Yu et al., 2019	++	++	*++*	*++*	*+++*	*++*	Rhesus Monkeys	Receptor Autoradiography	Lidow and Rakic, 1992
		+++	++	++	Mice	Genetic labeling	Wei et al., 2018									
	DRD3 */ Drd3*	?	?	++	Mice	Genetic labeling	Li and Kuzhikandathil, 2012									
	DRD4 */ Drd4*	?	++	++	Mice	Genetic labeling	Noaín et al., 2006	(+)	*++*	*+*	*++*	*+++*	*+++*	Humans	*In situ*	Weickert et al., 2007

*L, cortical layer; +++ highest expression; ++ intermediate expression; + low expression; (+) absent/very low expression.*

Taking into account these methodological limitations, studies on DRD proteins and their transcripts indicate that of the five DRD subtypes, DRD1 and its mRNA are most highly expressed in the adult rodent PFC, followed by DRD2/*Drd2*. In comparison, DRD3, 4 and 5 show limited expression (Tarazi and Baldessarini, 2000; Lidow et al., 2003; Araki et al., 2007; Rajput et al., 2009; Santana et al., 2009). DRD1 and DRD2 are expressed in both pyramidal neurons and interneurons of rodent PFC but are rarely colocalized (Santana et al., 2009; Zhang et al., 2010). RNA in situ hybridization studies in adult rats show that cells expressing *Drd1* mRNA are most prominent in layer VI, extending into layer V, with an additional thin band of positive cells in layer II. *Drd2*-expressing cells are mainly localized in layer V and VI, with few positive cells in layer II and III (Gaspar et al., 1995; Santana and Artigas, 2017). This laminar distribution pattern of DRD1 and DRD2 in rat mPFC was also observed in an earlier receptor binding study using fluorescently coupled receptor antagonists (Vincent et al., 1993). More recently, genetic labeling has emerged as an additional tool to monitor *Drd1-* and *Drd2*-expressing neurons in rodents. Genetic labeling studies involve transgenic mice that accommodate a BAC (bacterial artificial chromosome) construct containing *Drd1* or *Drd2* regulatory regions directing expression of Cre recombinase (*Drd1-Cre* or *Drd2-Cre* mice) (Gong et al., 2007). These Cre mice are crossed with reporter mice that express fluorescent proteins upon Cre-mediated recombination (such as *Ai14* or *Ai6* mice) allowing the identification of cells that express *Drd1* or *Drd2* (Madisen et al., 2010; Wei et al., 2018). In rats, *Drd2-Cre* knock-in animals have been generated and crossed with a fluorescent rat reporter line (*Ai9)* (Madisen et al., 2010; Yu et al., 2019). An important aspect to keep in mind with these Cre reporter systems is that recombination of the reporter allele is permanent, meaning that if the *Drd1* or *Drd2* promoter is transiently active in certain cell populations during embryonic or postnatal development, these cells will be recombined and continue to express the fluorescent protein in the adult brain even when these neuronal populations may no longer express *Drd1* or *Drd2* in the adult. Furthermore, in this system, the expression level of the fluorescent protein does not correspond to the level of endogenous gene or protein expression. Despite these caveats, in *Drd2-Cre, Ai9* reporter rats, the distribution of recombined cells (expressing fluorescent reporter protein) is largely in agreement with previous findings on *Drd2* expression in the mPFC (Santana and Artigas, 2017). Analysis of recombined cells in the anterior cingulate cortex show them mostly to be putative pyramidal neurons of upper and deep layers. Only a small number of inhibitory interneurons exhibit fluorescent labeling in this region (Yu et al., 2019). Similarly, in *Drd1-Cre, Ai6 or Drd1-Cre, Ai14* reporter mice, fluorescently labeled cells show a laminar distribution comparable to what has been reported for *Drd1* transcript expression in mPFC, with a higher overall density of *Drd1* expression in deep layers. In *Drd2-Cre Ai6/Ai14* mice, however, distribution of fluorescently labeled cells in mPFC is strikingly distinct from the one reported in *Drd2-Cre, Ai9* reporter rats or the expression patterns observed in RNA in situ hybridization studies, showing high expression of *Drd2* in superficial layers rather than in deep layers (Wei et al., 2018). Whether this is due to the different approaches used to generate the Cre-lines (BAC transgenic mice versus knock-in rats) or reflects a transient expression of *Drd2* in superficial layers of the mPFC during development in the mouse is unclear (Beil et al., 2012; Yu et al., 2019). BAC transgenic mice expressing enhanced green fluorescent protein (EGFP) under the transcriptional regulation of *Drd3* (*Drd3*-*Egfp* mice) or *Drd4* (*Drd4*-*Egfp* mice) locus have also been used to study the expression of *Drd3* and *Drd4* in different regions of the brain (Gong et al., 2003). In the *Drd3*-*Egfp* mouse model, the fluorescent cells in the caudal cingulate cortex are mainly located in layer VI (Li and Kuzhikandathil, 2012). Analysis of *Drd4-Egfp* mice showed strongly labeled EGFP-expressing neurons in layer V and VI of prelimbic and cingulate cortices (Noaín et al., 2006). DRD5 immunoreactivity has been detected in layer II to layer VI of prelimbic and cingulate cortices, with more labeled cells in layer II and III. In mice, DRD5 is more uniformly distributed across the cortical layers of the mPFC (Ciliax et al., 2000; Lidow et al., 2003; Table 1).

The developmental time course of DRD expression in rodent PFC is not well characterized and appears to vary considerably between rats and mice. RT-qPCR analysis in the murine cingulate cortex (both at rostral and caudal levels) at P0, P21, and P60 reveals that other than *Drd4*, which has the highest expression at birth followed by a rapid postnatal decrease in expression, transcript levels of the *Drd* subtypes do not show any significant developmental change between P0 and P60 (Araki et al., 2007). In the frontal cortex of rats, in situ hybridization signals for *Drd1* or *Drd2* transcripts have been detected around E14 or E18, respectively (Schambra et al., 1994). According to the same study, expression levels for both *Drd1* and *Drd2* appear to reach maximal levels between P14 and P30, although the change in signal intensity has not been quantified. Another study, however, shows that *Drd1, Drd5, Drd4, Drd2l* (but not *Drd2s*) expression in the mPFC of rats reaches peak expression only at P45 and then decreases between P45 and P70 (Naneix et al., 2012). At the protein level, there is a marked decline of DRD1 and DRD2 density in PFC of rats between adolescence (P40) and adulthood (P120) (Andersen et al., 2000). An earlier study using quantitative autoradiography in rats has described a similar pattern for DRD1 in mPFC, but with peak receptor binding density at P14 and P21, and a decrease in binding between P21-P42 (Leslie et al., 1991). A certain population of mPFC pyramidal neurons projecting to the nucleus accumbens also shows differential expression of DRD1 across postnatal development. In retrogradely traced prelimbic pyramidal neurons projecting to the nucleus accumbens core, the number of DRD1 immunoreactive cells was significantly higher in adolescents (P44) than in juveniles (P27) or adults (P105) (Brenhouse et al., 2008). Tarazi and Baldessarini, however, report a different temporal expression pattern in frontal cortex of rats. In their investigation, binding of radioligands to DRD1, DRD2 and DRD4 receptors gradually rises from P7 to maximal levels at P60 (Tarazi and Baldessarini, 2000). Overall, the data from various published studies do not deliver a conclusive picture on the time course and distribution of DRD/*Drd* expression in the developing mPFC (Table 2).

**TABLE 2:** Relative changes in expression of *Drds*/DRDs in PFC throughout postnatal development.

	Receptor / Gene	Rodent								Human / Non-human Primate									
		0W	1W	3W	6W	9W	Species	Method	References	S1	S2	S3	S4	S5	S6	S7	Species	Method	References
D1 – like Family	DRD1 */ Drd1*	+		↔		↔	Rats	RT-qPCR	Araki et al., 2007	+	↓			↑	↔	↓	Humans	*In situ*	Weickert et al., 2007
				+	↑	↓*	Rats	*In situ*	Naneix et al., 2012	+	↑	↑	↑	↔	↓	↓	Humans	RT-qPCR + Microarray	Rothmond et al., 2012
			+	↑*	↑	↑	Rats	Receptor Autoradiography	Tarazi and Baldessarini, 2000	*+*	↑	↑	↔	↑	↑*	↔	Humans	Western Blot	Rothmond et al., 2012
			+	↑	↓		Rats	Receptor Autoradiography	Leslie et al., 1991	+	↔	↑*	↓	↓	↔	↔	Rhesus Monkeys	Receptor Autoradiography	Lidow and Rakic, 1992
	DRD5 */ Drd5*	+		↔		↔	Rats	RT-qPCR	Araki et al., 2007	+	↔	↔	↔	↔	↔	↔	Humans	RT-qPCR + Microarray	Rothmond et al., 2012
				+	↑	↓*	Rats	RT-qPCR	Naneix et al., 2012										
D2 – like Family	DRD2 */ Drd2*	+		↔		↔	Rats	RT-qPCR	Araki et al., 2007	+	↓*			↑	↓	↑	Humans	*In situ*	Weickert et al., 2007
	*Drd2l*			+	↑	↓*	Rats	RT-qPCR	Naneix et al., 2012	+	↓	↑	↓	↓	↔	↔	Humans	RT-qPCR	Rothmond et al., 2012
	*Drd2s*			+	↔	↔	Rats	RT-qPCR	Naneix et al., 2012	+	↓	↔	↓*	↔	↔	↔	Humans	RT-qPCR	Rothmond et al., 2012
			+	↑*	↑	↑	Rats	Receptor Autoradiography	Tarazi and Baldessarini, 2000	+	↔	↑*	↓	↔	↔	↔	Rhesus Monkeys	Receptor Autoradiography	Lidow and Rakic, 1992
	DRD3 */ Drd3*	+		↔		↔	Rats	RT-qPCR	Araki et al., 2007										
	DRD4 */ Drd4*	+		↓*		↔	Rats	RT-qPCR	Araki et al., 2007	+	↔			↔	↔	↔	Humans	*In situ*	Weickert et al., 2007
				+	↑	↓*	Rats	RT-qPCR	Naneix et al., 2012	+	↔	↔	↔	↔	↔	↔	Humans	RT-qPCR	Rothmond et al., 2012
			+	↑*	↑	↑	Rats	Receptor Autoradiography	Tarazi and Baldessarini, 2000										

+ first postnatal stage analyzed & expression detected. ↑ increase; ↓ decrease; ↔ no change in expression compared to previous timepoint; * indicates increase or decrease in expression compared to previous timepoint that were statistically significant; empty cells: no data available. **W:** Week **S:** Stage; **S1**: neonate in humans, 0 month in rhesus monkeys; **S2**: infant in humans, 1 month in rhesus monkeys; **S3**: toddler in humans, 2 months in rhesus monkeys; **S4**: school age in humans, 8 months in rhesus monkeys; **S5**: adolescent in humans, 12 months in rhesus monkeys; **S6**: young adult in humans, 36 months in rhesus monkeys; **S7**: adult in humans, 60 months in rhesus monkeys.

An additional potent tool to investigate the distribution of *Drd* transcripts is single-cell mRNA sequencing (scRNA seq). DropViz is an extensive collection of scRNA seq data, assembled from analysis of RNA expression of thousands of individual cells across different regions of mouse brain (P60–P70) (Macosko et al., 2015; Saunders et al., 2018). Based on gene expression profiles in the frontal cortex (including the mPFC, orbital cortices, frontal association cortex, anterior parts of primary and secondary motor cortices, insular cortex and somatosensory cortex), *Drd1* and *Drd5* expression is highest in deep layer pyramidal neurons and *Drd4* is mostly expressed in pyramidal cells of layer II/III. *Drd2* transcript levels are notably low and are predominantly found in interneurons rather than in projection neurons. Additionally, a rather remarkable observation is that the highest level of *Drd1* and *Drd2* expression is found in microglia. *Drd3* expression is not included in this transcriptional analysis of the frontal cortex, possibly because of low expression levels. The transcriptional dynamics of the *Drds* in the frontal cortex during development has not yet been investigated. However, dynamic regulation of *Drd1* has been demonstrated in the context of mouse models of drug abuse. Bhattacherjee and colleagues have shown that chronic cocaine addiction induces cell type-specific transcriptional changes in the murine mPFC. The effect of cocaine addiction on gene expression changes was particularly striking during the withdrawal period, with excitatory neurons in the deeper layers being more affected. While the most significantly affected excitatory clusters expressed *Drd1*, the analysis also detected *Drd1* expressing excitatory clusters that did not respond robustly to cocaine. Although the functional role of each subtype remains to be investigated, this suggests that certain *Drd1*-expressing neuronal subtypes in the PFC may be more involved in the process of cocaine addiction than others (Bhattacherjee et al., 2019). Further analysis on dataset of cocaine-addicted mice revealed that *Drd1* and *Drd2* genes are both upregulated in cocaine addiction and are almost solely expressed in excitatory neurons, with *Drd1* also being found at lower levels in inhibitory neurons, oligodendrocyte and endothelial cells in the mPFC (Bhattacherjee et al., 2019; Navandar et al., 2021).

In addition to DRD expression patterns, maturation of receptor function could also contribute to changing impact of the mesoprefrontal system over time. Investigations into DRD function have shown that DRD1-mediated modulation of NMDA receptor transmission prompt recurrent depolarizing plateaus in pyramidal neurons of mPFC slices, an effect that develops only after P45 (Tseng and O’Donnell, 2005). Furthermore, DRD2-mediated increase in excitability of fast-spiking interneurons in PFC slices appears only after P50 (Tseng and O’Donnell, 2007). Thus, while the changes in postnatal expression levels of DRD/*Drd* are still unclear, there is indeed a change in activity of DRDs in the post-pubertal stage, hinting towards the role of DA in the remodeling of PFC microcircuits during the transition from adolescence to adulthood.

Another gap in our understanding of DRD receptor expression and function in the developing and adult mPFC is that we know little about the subcellular localization of receptors in DRD-expressing neurons. Because existing antibodies against DRDs have limited utility for detecting DRDs in brain tissue (Bodei et al., 2009), alternative approaches should be considered for investigating this question. Vincent and colleagues analyzed cellular localization of D1- and D2-like family of receptors in the mPFC using receptor antagonists coupled to fluoroprobes and observed that around 25% of all fluoroprobe-labeled cells displayed both D1 and D2-like subfamily receptor binding fluorescence along the outer edge of the soma. Further analysis on cell size distribution suggested that the cells in which colocalization could be detected were non-pyramidal (Vincent et al., 1995). A recent promising technique to examine subcellular localization of DRDs may be the application of CRISPR/Cas9 based genome editing tools to introduce fluorescent tags to endogenous receptor proteins. A recent study used a modified CRISPR/Cas9 knock-in strategy with two guide RNAs to knock-in a fluorescent protein to α-amino-3-hydroxy-5-methyl-4-isoxazolepropionic acid (AMPA) and N-methyl D-aspartic acid (NMDA) receptor subunits in primary mouse cortical cultures (Fang et al., 2021). The application of these epitope tags in vivo is also possible. Using the so-called ORANGE (Open Resource for the Application of Neuronal Genome Editing) toolbox, adeno-associated virus plasmids containing fluorescent tag knock-in constructs for PSD95 and AMPA receptor subunit (GLUA1) were injected into the hippocampus of Cas9-P2A-GFP transgenic mice resulting in robust labeling of both proteins (Willems et al., 2020). Applying these methods for the fluorescent tagging of DRDs has the potential to aid in determining the subcellular localization of DRDs in fixed tissue as well as monitoring dynamics of receptor localization in dissociated cell cultures in vitro or in acute slices.

The distribution of DRDs in the rodent mPFC correlates largely with the innervation pattern of DA fibers, suggesting that DRD expression might be influenced by DA release in the mPFC. In this context, DA might play a role during the phase when projections are established (as in a critical developmental period) and/or influence DRD expression levels in the adult brain. Indeed, there is evidence from the striatum that ablating DA innervation during early postnatal development (using 6-OHDA-mediated lesion of the nigrostriatal and mesolimbic pathway at neonatal stages) results in reduced binding of radioligand to DRD1 in the caudate putamen and nucleus accumbens of the adult (P90) rat. Radioligand binding to DRD2 is not affected (Thomas et al., 1998). In the adult brain, the loss of striatal DA input in Parkinson’s disease patients or in animal models of the disease leads to compensatory upregulation of DRDs, while drug-induced DA increase in the nucleus accumbens leads to reduced expression of DRDs to adjust for elevated DA in the system (Hisahara and Shimohama, 2011; Volkow and Morales, 2015). In the mPFC, the influence of DA on DRD expression has not been studied in detail. One study has examined the effect of depletion of DA projections in the postnatal rat by intracisternal injection of 6-OHDA 5 days after birth and found that DRD1 receptor binding remains unaltered (Leslie et al., 1991). Mouse models interfering with the development of mesoprefrontal projections, such as the *Dcc* and *Netrin-1* haploinsufficient mice that elevate DA transmission in the mPFC (Vosberg et al., 2020) or mouse models that lack mesoprefrontal innervation (Kabanova et al., 2015) may offer a suitable approach to determine the role of DA innervation in the developmental trajectory of DRD expression in the mPFC.

### Dopamine Receptor Expression in Primate Prefrontal Cortex

In the adult PFC of rhesus monkeys, autoradiographic receptor binding assays showed that DRD1 is most densely present in layers I, II, IIIa, V, and VI, while DRD2 shows the highest expression density in layer V of adult PFC (Lidow and Rakic, 1992) (Table 1). Immunohistochemistry for DRD1 and DRD5 in rhesus monkey PFC (area 9) demonstrated that these receptors widely colocalize on spines of pyramidal neurons and axon terminals (Bordelon-Glausier et al., 2008). In the adult human PFC, DRD1, DRD2 and DRD4 are highly expressed in deeper layers (layer V and VI) and layer II (Weickert et al., 2007; Table 1). These studies did not report on the expression of DRD3.

DRDs appear to be dynamically expressed in the developing PFC in primates. In the adult rhesus monkey PFC (5–6 years old), DRD1 and DRD2 density (examined by autoradiographic receptor binding assays) was found to be significantly lower compared to 2 months of age (Lidow and Rakic, 1992). Another study, using [^11^C] FLB 457 (high-affinity radioligand for DRD2/3) in positron emission tomography (PET) on human subjects (age range 19–74 years), detected a significant decline in DRD2/3 expression with age in the frontal cortex area (Kaasinen et al., 2000). An immunohistochemistry study was not performed for these receptors. At the transcriptional level, a cohort study of human post-mortem PFC tissue revealed that *DRD1* mRNA is expressed at neonatal stages. Expression levels decline within the first year of life, are highest during adolescence and young adults, and gradually decline again in adult and aged cohorts (Weickert et al., 2007). However, in a similar cohort study of the human dorsolateral PFC, *DRD1* mRNA expression was reported to increase steadily until adolescence but to decrease slightly thereafter. Western blot analysis of DRD1 expression indicated that protein levels also increase gradually with age, but the highest expression was found in the young adult and adult groups (Rothmond et al., 2012). Moreover, a similar layer-specific pattern was observed across all studied ages: *DRD1* transcript levels were not detected in layer I of the human dorsolateral PFC, were present at an intermediate level in layers III and IV and highest expression was found in layers II, V, and VI (Weickert et al., 2007). Unlike *DRD1, DRD2* expression levels peak at neonatal age, followed by a significant decrease in infants. At all later developmental time points examined, expression levels remain below neonatal levels. Similarly, mRNA levels of the short (*DRD2S*) and long (*DRD2L*) *DRD2* isoform are highest at the neonatal stage and decrease with age in the dorsolateral PFC. A layer-specific pattern was observed also for *DRD2* with highest expressions in layers II, V, and VI. *DRD1* and *DRD2* mRNA was found in both pyramidal and non-pyramidal neurons in adult brain (Weickert et al., 2007). *DRD4* mRNA expression was detected in presumed non-pyramidal neurons and glia but was barely present in pyramidal cells (Weickert et al., 2007). Generally, *DRD4* did not show any age-specific changes in expression and highest signal intensity was detected in layer V (Weickert et al., 2007; Rothmond et al., 2012). *DRD5* expression levels did not show any significant differences between age groups (Rothmond et al., 2012). To the best of our knowledge, the distribution of *DRD3* expression in the developing primate PFC has not yet been reported (Table 2).

Similar to what we have highlighted above for the investigation of *Drd* expression in the rodent brain, high-throughput techniques for transcriptome analysis, such as scRNAseq, give now the opportunity to explore the cell-type specific expression of *DRD* transcripts in the developing and adult human PFC in further detail (Allen Institute for Brain Science, 2010; Fan et al., 2018, 2020; Zhong et al., 2018; Polioudakis et al., 2019; Tanaka et al., 2020; Maynard et al., 2021). This will be instrumental in defining temporal dynamics and cell type-specific responsiveness to DA.

Finally, neither DA release nor receptor expression may offer a full reflection of how DA impacts on cortical neurons in the PFC. As discussed above, DRDs act on DA-receiving cells by modulating PKA activity. Thus, monitoring PKA activity may offer additional insight into the effects of DA on cortical neuronal function. A recent study used a PKA activity sensor to monitor the effect of DA release on *Drd1*- versus *Drd2*-expressing medium spiny neurons in the nucleus accumbens during learning in real-time (Lee et al., 2021). However, given that DA innervation and release is much sparser in the mPFC than in the nucleus accumbens and other modulatory neurotransmitters released in the mPFC (e.g., NA, Serotonin) act also via G-protein coupled receptors and modulation of PKA activity, further studies would be needed to determine whether a similar approach could be applied in the PFC.

In summary, a better understanding of the developmental time course of DA release; the laminar distribution, neuronal subtype expression, and subcellular localization of DRD receptors as well as downstream signaling events would greatly contribute to our knowledge of the functional role of DA in the developing and adult PFC.

## The Developing Mesoprefrontal System in Neuropsychiatric Diseases

As discussed above, the PFC is the region of the brain that is particularly important for executive functions and the control of goal-directed and self-regulatory behaviors. Dysregulation of local micronetworks in the PFC has been associated with impaired social, affective, and cognitive functions typically seen in neurodevelopmental disorders such as schizophrenia, autism spectrum disorder and attention deficit/hyperactivity disorder as well as in depression and substance abuse disorders. An open question is to what extent deficits in the mesoprefrontal DA system, and thus DA-influenced neuromodulation of local PFC networks, contribute to the pathophysiology of these neuropsychiatric disorders. In particular, it is unclear whether these changes occur secondary to alterations in the PFC (and other cortical areas) or can also be attributed to developmental deficits in the mesoprefrontal DA system. Many of the mutations associated with schizophrenia or autism spectrum disorder are found in genes encoding synaptic proteins. While loss of function of these genes has been shown to lead to deficits in synaptic transmission in cortical regions and particularly in the PFC, it is not known whether this also directly affects the function of mesoprefrontal DA neurons (Yan and Rein, 2021). Another point that should be considered in this context, is that mesoprefrontal DA neurons (at least in rodents) can co-release glutamate (Kabanova et al., 2015; Mingote et al., 2015; Pérez-López et al., 2018; Zhong et al., 2020). Thus, any developmental deficits or alterations in the mesoprefrontal system could have consequences for both DA and glutamate release in the PFC. In the following, we will focus on the possible dysfunction of the mesoprefrontal system in three neuropsychiatric diseases with a clear developmental etiology: schizophrenia, autism spectrum disorder, and attention deficit/hyperactivity disorder. In the context of these diseases, we will briefly discuss a few studies that have examined potential alterations in the developing DA system.

### Schizophrenia

Schizophrenia is a neuropsychiatric disorder with severe symptoms that usually become manifest in full during adolescence or early adulthood. These include the so-called positive symptoms (psychosis), negative symptoms (deficits in emotional responses and thought processes), and cognitive dysfunction (e.g., deficits in working memory, long-term memory, semantic processing, learning) (Marder and Cannon, 2019). According to the so-called DA hypothesis of schizophrenia, alterations in DA signaling are a major factor in these disease symptoms: DA hyperactivity in the striatum promotes psychosis, while DA hypoactivity in other brain areas, including the PFC, contributes to the negative symptoms and cognitive dysfunction. There is ample evidence from human studies to support this hypothesis. To name a few: (1) DA agonists and stimulants such as cocaine or amphetamine can induce psychosis in healthy individuals and exacerbate psychosis in patients with schizophrenia; (2) antipsychotic drugs act on the DA system via DRD2 receptors (e.g., haloperidol); (3) postmortem studies have demonstrated increased levels of DRDs, DA, and DA metabolites in the striatum of patients with schizophrenia; (4) imaging studies in patients with schizophrenia show that stimulant-induced presynaptic DA release is decreased in most brain regions, except for the striatum, where it is increased. For further details, we refer the interested reader to a collection of reviews on the DA hypothesis of schizophrenia (Biol Psychiat, 2017).

With respect to the mesoprefrontal DA system, its hypoactivity is most likely associated with the cognitive dysfunctions in schizophrenia. The cause of the overall DA imbalance may be caused by deficits in local cortical or hippocampal networks that in turn lead to changes in the inputs to the VTA from these regions and ultimately to DA hypoactivity in VTA targets. Alternatively, or in addition, defects in the regulation of DA release in target regions (including the PFC) or in the developmental of the mesoprefrontal system could contribute to the DA hypoactivity (Rice et al., 2016; Abi-Dargham, 2017; Chuhma et al., 2017; Grace, 2017; Walker et al., 2017; Sonnenschein et al., 2020; Braun et al., 2021). Whether the development of the mesoprefrontal DA system (or other parts of the DA system) is altered in patients with schizophrenia has not yet been studied in detail.

### Autism Spectrum Disorders

Autism spectrum disorder (ASD) encompasses a group of severe neurodevelopmental disorders that exhibit core symptoms of social and communication deficits and stereotyped, repetitive behaviors (Association, 2013; Fein et al., 2021). Many studies highlight similar behavioral and cognitive impairments between ASD and schizophrenia such as social and language deficits and there is a high co-occurrence of both neurodevelopmental disorders (Spek and Wouters, 2010; King and Lord, 2011; Chisholm et al., 2015; Crescenzo et al., 2019). Based on this, it has been speculated that dysfunction in the DA system may also contribute to the cognitive disorders in ASD and, similar to schizophrenia, a DA hypothesis has been proposed for ASD. According to this hypothesis, aberrant mesocorticolimbic and nigrostriatal DA circuitry may contribute to reward deficits and goal-directed motor impairments manifested in ASD children (Pavăl, 2017; Pavăl and Micluţia, 2021). Initial evidence for impairments in the DA system in ASD came from a study that found elevated levels of DA metabolites, such as homovanillic acid, in the cerebrospinal fluid of autistic children (age 1- 16 years old) (Gillberg and Svennerholm, 1987). Further evidence supporting this hypothesis comes from (1) the discovery that de novo genetic variants of the gene encoding the dopamine transporter (*DAT)* (Neale et al., 2012; Hamilton et al., 2013; Bowton et al., 2014; Cartier et al., 2015) and gene polymorphisms in *DRD3* and *DRD4* (Gadow et al., 2010; Staal, 2014; Staal et al., 2015) are associated with ASD; (2) the therapeutic efficacy of DRD blockers (risperidone and aripiprazole) in alleviating stereotypic and/or abnormal social behaviors in children with autism (McCracken et al., 2002; McDougle et al., 2005; Ghaeli et al., 2014) and (3) studies showing that the reward circuitry is hypoactivated in autistic patients in response to social and monetary rewards (Zeeland et al., 2010; Dichter et al., 2012; Kohls et al., 2012). According to the DA hypothesis in ASD, this diminished ability to register rewards for social cues could lead to the decreased pursuit of social interaction and ultimately to the deficits in social and communication skills observed in ASD patients (Pavăl, 2017). Regarding the mesoprefrontal system, an early PET scanning study for fluorine-18-labeled fluorodopa (F-DOPA) revealed significantly decreased F-DOPA ratio in the anterior mPFC of autistic children compared to healthy subjects, indicating decreased DA activity in the mPFC in autistic patients (Ernst et al., 1997). ASD patients underperform in working memory tasks involving planning, cognitive flexibility, and high working memory load compared to control subjects, which could be due to, or at least influenced by, a dysfunctional mesoprefrontal DA system (Kercood et al., 2014). Moreover, computational models predict that decreasing DA modulation in the PFC could lead to executive dysfunctions such as decreased cognitive flexibility, as occurs in ASD (Kriete and Noelle, 2015). Nevertheless, it remains largely unclear whether impairments of the mesoprefrontal DA system contribute to cognitive deficits in ASD patients and whether the development of mesoprefrontal mDA neurons is altered in ASD. The phenotypic heterogeneity of ASD and largely unknown disease mechanisms complicate the investigations of these potential deficits.

To uncover the potential role of altered development of the DA system and in particular the mesoprefrontal DA neurons in ASD etiology and associated social and executive dysfunctions, further DA system-focused studies in patients and ASD mouse models are needed. Evidence from mouse models for the involvement of the DA system in ASD is discussed in detail in a recent review (Kosillo and Bateup, 2021), thus we will only discuss two examples here. Mutations in the gene encoding SH3 and multiple ankyrin repeat domains 3 (SHANK3), a postsynaptic scaffolding protein, have been discovered in ASD patients, making it a prominent autism gene candidate (Gauthier et al., 2009; Phelan and McDermid, 2012; Boccuto et al., 2013). Studies on the *Shank3* haploinsufficient mouse model show that impaired preference for social interactions is due to decreased DA activity in the VTA (Bariselli et al., 2016, 2018). Whether this hypoactivity results in decreased DA release in the nucleus accumbens and/or the mPFC has not yet been addressed. A potential link between autistic-like phenotypes and aberrant development of the DA system emerges from animal models for Mucopolysaccharidosis (MPS). MPS are hereditary lysosomal storage diseases, in which dysfunctions in lysosomal hydrolases lead to the accumulation of undegraded glycosaminoglycans in lysosomes and eventually to disturbances in cellular metabolism. In MPS IIIa, in which the gene coding for the lysosomal hydrolase sulfamidase is mutated, the metabolic cellular deficits result in neurodegeneration and dementia in children. Dementia is preceded by severe autistic-like behaviors (Valstar et al., 2010; Rumsey et al., 2014). In a mouse model of MPS IIIa, inactivation of the gene coding for sulfamidase, results in severely impaired behavior that that can be considered autism-like. These behavioral deficits are associated with increased DA release in the dorsal and ventral striatum and can be ameliorated with a DRD1 antagonist. This hyperdopaminergic state in MPS IIIa mice appears to be caused by developmental changes in the DA system: increased proliferation of mDA progenitors results in an increased number of mDA neurons in the SNpc and the VTA in the adult brain. Moreover, the same study shows that autistic-like behaviors and increased DA cell number are also present in a mouse model for a different type of MPS (MPS-II) (Risi et al., 2021). While this study suggests that altered development of the mDA system may be one of the causes of autism-like behaviors, it has not been investigated whether the increase in VTA neurons in these animal models leads also to alterations in the mesoprefrontal DA system. Further investigation of existing and potentially novel ASD candidate genes in animal models will be necessary to uncover developmental, structural, and/or functional impairments of the mesoprefrontal DA system in association with ASD.

### Attention Deficit Hyperactivity Disorder

Attention deficit/hyperactivity disorder (ADHD) is a highly heritable, early-onset neurodevelopmental disorder, characterized by symptoms of hyperactivity, short attention span, and impulsivity. The PFC is a key region afflicted in this disorder. Studies report thinning of PFC areas, reduced density of the dorsolateral PFC, and decreased PFC activity in ADHD patients compared to controls (Arnsten and Pliszka, 2011; Cortese, 2012; Klein et al., 2019). Shaw and colleagues reported that the PFC in children with ADHD takes significantly longer to reach peak cortical thickness compared to the PFC in typically developing individuals, suggesting a delay in PFC maturation (Shaw et al., 2007a). The typical ADHD symptoms also reflect impaired executive functioning of PFC, which in turn is related to dysregulated NA and DA signaling in the PFC (Arnsten and Pliszka, 2011). There are several points of evidence that suggest that alterations in the DA system may contribute to ADHD symptoms. An F-DOPA PET study showed low DOPA-decarboxylase activity in the PFC of adult ADHD patients compared to healthy controls, an effect that could however not be replicated in adolescents with ADHD (Del Campo et al., 2011). Methylphenidate and amphetamine, which are used in the treatment of ADHD, act by inhibiting DA and NA reuptake and consequently by increasing DA and NA transmission in the PFC. Low doses of methylphenidate have been shown to improve PFC function in rats and monkeys, which can be counteracted by blocking DRD1 receptor. Moreover, mice heterozygous for the gene encoding dopamine transporter (DAT hypofunction mice), show behavior typical for ADHD such as hyperactivity, inattention, and impulsivity. Inattentive and impulsive behavior in these mice can be rescued by amphetamine. In humans, using radiolabeled altropane, a high-affinity selective probe for DAT, neuroimaging studies point towards evidence of increased DAT activity in striatum of children and adults with ADHD. However, due to its limited expression, it has been challenging to analyze DAT levels in the cortex using PET imaging techniques and it is still poorly characterized in the PFC of ADHD patients (Spencer et al., 2005; Prince, 2008). In addition, there is a significant association between ADHD and polymorphism in the genes that encode DRD4, DRD5, and DAT. *DRD4* has a high number of polymorphisms in its nucleotide sequence. Comprehensive meta-analyses showed that the so-called *DRD4* 7-repeat allele (*DRD4* 7R; a 7-repeat form of the 48–base pair (bp) variable number tandem repeat) elevates the risk of ADHD (Wu et al., 2012). Shaw and colleagues showed that presence of *DRD4* 7R was linked to cortical thinning in orbitofrontal and inferior prefrontal cortex that was augmented in ADHD patients (Shaw et al., 2007b). Another study suggests a considerable reduction in gyrification of inferior frontal gyrus in children with ADHD, who were *DRD4* 7R allele carrier. The authors hypothesize that this *DRD4* polymorphism could affect early stages of cortical development in children who later develop ADHD (Palaniyappan et al., 2019). Additionally, a 148-bp and a 136-bp dinucleotide repeat allele from the *DRD5* gene have also received considerable attention while the most extensively studied *DAT* polymorphism involves the 40 bp 9-repeat and 10-repeat alleles (Gizer et al., 2009; Wu et al., 2012).

An evolutionary perspective on ADHD argues for an adaptive role of the mesoprefrontal system in the disorder. Symptoms associated with ADHD, such as hyperactivity or limited sustained attention, could help animals to detect threats more rapidly and hence serve as beneficial features in endangered situations (Jensen et al., 1997; Lee and Goto, 2015). When delayed PFC maturation puts animals at a disadvantage in an adverse environment, ADHD symptoms arising from reduced mesoprefrontal DA could emerge as a compensative mechanism to make animals less vulnerable to the environmental threats. While such an adaptive response may have aided ancestral humans in stressful conditions, it does not translate well to modern social settings (Lee and Goto, 2015).

In summary, these data indicate that changes in DA signaling, in particular in the PFC may play a critical role in the pathophysiology of ADHD. However, it remains challenging to separate the impact of altered DA versus NA signaling on PFC dysfunction in ADHD. It also should be taken into consideration, that similar to schizophrenia and ASD, alterations in DA signaling could be secondary to functional changes in cortical areas (Arnsten and Dudley, 2005; Gamo et al., 2010; Arnsten and Pliszka, 2011; Mereu et al., 2017). The etiology of ADHD is multifaceted, having a strong genetic background but also contributions from environmental risk factors. Beside PFC, other brain regions having reciprocal connection to PFC, such as caudate and cerebellum are affected and there is an intricate interplay of neurotransmitters distinctive to each region (Arnsten and Pliszka, 2011; Cortese, 2012). Our understanding of the role of reduced mesoprefrontal signaling among these complex interactions is still evolving (Stanford and Heal, 2019) and requires further studies to better understand both, its specific function, and its complementary role along with DA signaling in the subcortical brain regions, in the pathophysiology of ADHD.

## Conclusion

Research over the past decade has vastly increased our knowledge of the development of mDA neurons and their molecular and functional diversity. Despite these advances, fundamental questions about the development and function of the mesoprefrontal DA system remain unresolved. For example, it is still unclear whether mesoprefrontal mDA neurons arise from a specific mDA progenitor population during development and whether these neurons can be defined at the molecular level as a specific mDA subset. Findings on the developmental history and molecular profile of these neurons would facilitate specific manipulation of the mesoprefrontal DA system by genetic methods (e.g., optogenetics, chemogenetics). This would allow to examine the consequences of functional changes in mesoprefrontal DA release on PFC development and PFC-regulated behavior. A possibility to specifically study the mesoprefrontal system during development and in the adult brain would most likely also provide further insights into a potential causative role of mesoprefrontal dysfunction in neurodevelopmental and neuropsychiatric disorders. Finally, how the mesoprefrontal system affects the activity of micronetworks in the PFC is still an open question, as it is still not fully understood at which stages, in which cell types and cortical layers DRDs are expressed in PFC and how DA release is coordinated with co-release of glutamate.

## Author Contributions

KI, NM, and SB: writing—original draft and review and editing. All authors contributed to the article and approved the submitted version.

## Conflict of Interest

The authors declare that the research was conducted in the absence of any commercial or financial relationships that could be construed as a potential conflict of interest.

## Publisher’s Note

All claims expressed in this article are solely those of the authors and do not necessarily represent those of their affiliated organizations, or those of the publisher, the editors and the reviewers. Any product that may be evaluated in this article, or claim that may be made by its manufacturer, is not guaranteed or endorsed by the publisher.
