# Supplementary material for: The Development of the Mesoprefrontal Dopaminergic System in Health and Disease
Source: Front Neural Circuits. 2021 Oct 12;15:746582. doi: 10.3389/fncir.2021.746582 (PMC8546303; doi:10.3389/fncir.2021.746582)
Supplement: Supplementary file 1 [file Image_1.pdf]

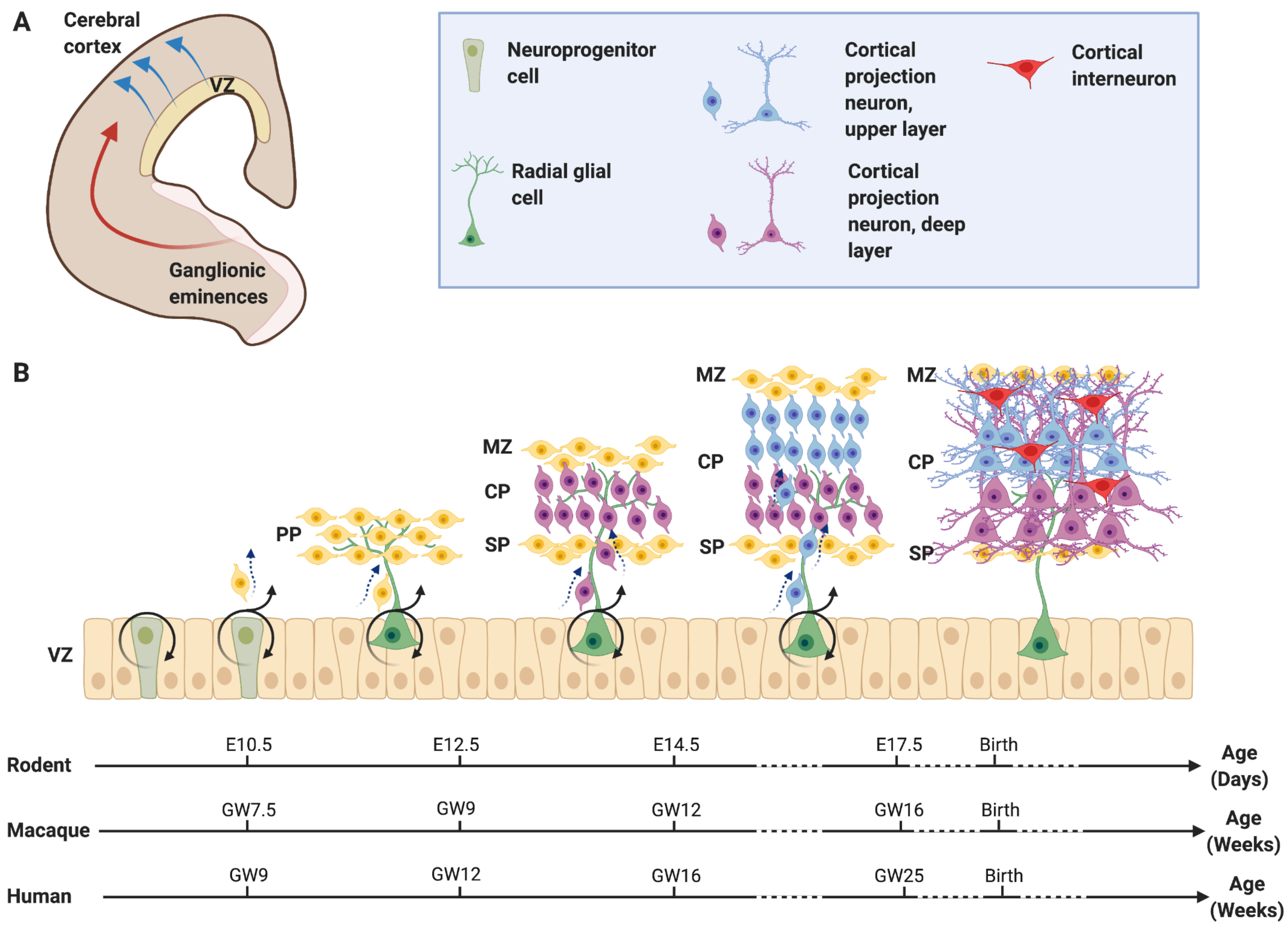

Supplemental Figure 1: Development of the cerebral cortex. Cortical projection neurons are generated in the ventricular zone (VZ) of the cortical plate (A, B) and migrate radially (blue arrows in A, B) along fibers of radial glia cells (green cells in B) until they reach the cortical pial surface (not shown), where they coalesce to form distinct cortical cell layers. The earliest born neurons that leave the VZ form the preplate (PP, yellow cells). Neurons born slightly later (purple), migrate into the PP, splitting it into the subplate (SP, yellow) and marginal zone (MZ, yellow) and start to form the cortical plate (CP). As cortical development proceeds, the newborn neurons migrate through the preformed layers of the earlier-born neurons. Thus, early-born projection neurons form the deep cortical layers (purple), while later-born neurons form the upper cortical layers (blue). In mice, deep layer neurons are born between embryonic day (E)12.5 and E13.5; upper layer neurons between E14.5 and E17.5 (Franco and Müller, 2013; Mukhtar and Taylor, 2018). Cortical interneurons are not generated from the VZ underlying the CP but are born in the ganglionic eminences in the ventral telencephalon (A), from where they migrate tangentially (red arrow in A) to the MZ of the CP (around E18.5 in mice). From the MZ, the cortical interneurons (red) migrate radially (not shown) to reach their final position within specific cortical layers (B). The final distribution within the cortical plate is distinct for different types of interneurons (not shown) (Wamsley and Fishell, 2017; Lim et al., 2018). The steps of cortical development in macaques and humans are similar in principle to those in rodents, but the neurogenic period is much longer, extending in humans into gestational week (GW) 25 (Lui et al., 2011; Mukhtar and Taylor, 2018, Clancy et al. 2001, Lewitus et al. 2014, Miškic et al. 2021) (B). During development, the primate cortex shows an additional zone containing cortical progenitors, the outer ventricular zone (not shown) (Lui et al., 2011; Mukhtar and Taylor, 2018). Similar to rodents, interneurons in primates are born in the ganglionic eminences (A) and use tangential followed by radial migration to reach their final position in the CP, a process that continues for several months after birth (Paredes et al., 2016; Kim and Paredes, 2021). Created with BioRender.com. Modified from the template: Embryonic Migration of Neuronal Progenitors Relies on Astrocytic Scaffold.
